# Analysis of Volatile Anesthetic-Induced Organ Protection in Simultaneous Pancreas–Kidney Transplantation

**DOI:** 10.3390/jcm11123385

**Published:** 2022-06-13

**Authors:** Nora Jahn, Maria Theresa Völker, Sven Laudi, Sebastian Stehr, Stefan Schneeberger, Gerald Brandacher, Elisabeth Sucher, Sebastian Rademacher, Daniel Seehofer, Hans Michael Hau, Robert Sucher

**Affiliations:** 1 Department of Anesthesiology and Intensive Care Medicine, University Hospital of Leipzig, Liebigstrasse 20, 04103 Leipzig, Germany; theresa.voelker@medizin.uni-leipzig.de (M.T.V.); sven.laudi@medizin.uni-leipzig.de (S.L.); sebastian.stehr@medizin.uni-leipzig.de (S.S.); 2 Department of Visceral, Transplant and Thoracic Surgery, Innsbruck Medical University, 6020 Innsbruck, Austria; stefan.schneeberger@i-med.ac.at; 3 Vascularized Composite Allotransplantation (VCA) Laboratory, Department of Plastic and Reconstructive Surgery, Johns Hopkins University, Baltimore, MD 21205, USA; gerald.brandacher@jhmi.edu; 4 Department of Oncology, Gastroenterology, Hepatology, Pneumology and Infectiology, University Hospital of Leipzig, 04103 Leipzig, Germany; elisabeth.sucher@medizin.uni-leipzig.de; 5 Department of Visceral, Transplantation, Vascular and Thoracic Surgery, University Hospital of Leipzig, 04103 Leipzig, Germany; sebastian.rademacher@medizin.uni-leipzig.de (S.R.); daniel.seehofer@medizin.uni-leipzig.de (D.S.); hans-michael.hau@uniklinikum-dresden.de (H.M.H.); robert.sucher@medizin.uni-leipzig.de (R.S.); 6 Department of Visceral, Thoracic and Vascular Surgery, University Hospital and Faculty of Medicine Carl Gustav Carus, Technische Universität Dresden, 01307 Dresden, Germany

**Keywords:** simultaneous pancreas–kidney transplantation, ischemia–reperfusion injury, volatile anesthetics, anesthesia, graft outcome, graft loss and graft function

## Abstract

Background: Despite recent advances in surgical procedures and immunosuppressive regimes, early pancreatic graft dysfunction, mainly specified as ischemia–reperfusion injury (IRI)—Remains a common cause of pancreas graft failure with potentially worse outcomes in simultaneous pancreas-kidney transplantation (SPKT). Anesthetic conditioning is a widely described strategy to attenuate IRI and facilitate graft protection. Here, we investigate the effects of different volatile anesthetics (VAs) on early IRI-associated posttransplant clinical outcomes as well as graft function and outcome in SPKT recipients. Methods: Medical data of 105 patients undergoing SPKT between 1998–2018 were retrospectively analyzed and stratified according to the used VAs. The primary study endpoint was the association and effect of VAs on pancreas allograft failure following SPKT; secondary endpoint analyses included “IRI- associated posttransplant clinical outcome” as well as long-term graft function and outcome. Additionally, peak serum levels of C-reactive protein (CRP) and lipase during the first 72 h after SPKT were determined and used as further markers for “pancreatic IRI” and graft injury. Typical clinicopathological characteristics and postoperative outcomes such as early graft outcome and long-term function were analyzed. Results: Of the 105 included patients in this study three VAs were used: isoflurane (n = 58 patients; 55%), sevoflurane (n = 22 patients; 21%), and desflurane (n = 25 patients, 24%). Donor and recipient characteristics were comparable between both groups. Early graft loss within 3 months (24% versus 5% versus 8%, *p* = 0.04) as well as IRI-associated postoperative clinical complications (pancreatitis: 21% versus 5% versus 5%, *p* = 0.04; vascular thrombosis: 13% versus 0% versus 5%; *p* = 0.09) occurred more frequently in the Isoflurane group compared with the sevoflurane and desflurane groups. Anesthesia with sevoflurane resulted in the lowest serum peak levels of lipase and CRP during the first 3 days after transplantation, followed by desflurane and isoflurane (*p* = 0.039 and *p* = 0.001, respectively). There was no difference with regard to 10-year pancreas graft survival as well as endocrine/metabolic function among all three VA groups. Multivariate analysis revealed the choice of VAs as an independent prognostic factor for graft failure three months after SPKT (HR 0.38, 95%CI: 0.17–0.84; *p* = 0.029). Conclusions: In our study, sevoflurane and desflurane were associated with significantly increased early graft survival as well as decreased IRI-associated post-transplant clinical outcomes when compared with the isoflurane group and should be the focus of future clinical studies evaluating the positive effects of different VA agents in patients receiving SPKT.

## 1. Introduction

Ischemia–reperfusion injury (IRI) is a major cause contributing to early graft injury, which ultimately results in severe patient morbidity and mortality in standard solid organ transplantation [1]. However, the reperfusion phase during surgery especially offers an auspicious window of opportunity to mitigate IRI and improve short- and long-term outcomes. In this context, volatile anesthetics (VA) have been shown to interfere with many pathophysiological mechanisms contributing to the injurious cascade of IRI [2].

Previous studies have demonstrated the beneficial effects of volatile anesthetics (VA) in alleviating reperfusion injury in kidney and liver transplantation. Furthermore, transplant data are in line with evidence gained in general cardiovascular and neurosurgical procedures, which involve comparable phases of organ ischemia and reperfusion [3,4,5,6,7,8]. However, to date, there is only scarce information available on the effects of VAs in simultaneous pancreas and kidney transplantation (SPKT). Their impact on early and long-term graft and patient outcomes are even less characterized [9].

The pancreatic allograft is exquisitely susceptible to injuries in the early reperfusion period, which, among other serious complications, leads to damaging effects on the grafts’ microvasculature as well as dysfunction in both the endocrine and exocrine pancreas [1,10,11]. In particular, IRI is one of the main reasons for islet cell injury, which may be aggravated by periprocedural obstacles such as prolonged cold storage or insufficient flushing of the graft before transplantation [1,10,12,13]. Following the cold ischemic phase accompanied by reduced metabolic activity, the reperfusion and renewed onset of aerobic metabolism after reperfusion of the graft is the main mechanism for IRI [6]. Despite advancing technologies such as machine perfusion instead of cold storage, as well as refinements of surgical procedures and immunosuppressive medications, early allograft failure induced by IRI remains a serious problem, especially in pancreas transplantation [14,15,16,17,18,19,20]. Based on previous studies, graft-related complications, including pancreatitis and graft thrombosis, are the main reasons for nonimmunological early graft loss [14,15,21]. Parameters that identify patients at high risk for the development of post-transplant complications and graft failure would assist in the management of these especially vulnerable subgroups. In this context, previous studies showed encouraging results using peak levels of serum lipase and C-reactive protein (CRP) during the first days after pancreas transplantation as potential IRI markers for graft damage [22]. Further evaluation of early indicators of IRI and a thorough investigation of cellular pathways contributing to local inflammation and reperfusion injury may help develop preventive and rescue treatment strategies for IRI in solid organ transplant recipients and thereby improve graft function and outcome [23].

One interesting possibility that may attenuate IRI by inducing biochemical changes in different tissues seems to be pharmacological conditioning [2,7]. Herein, the administration of VAs has been shown to be effective in providing cellular tolerance against IRI in various tissues and organs, including the heart, brain, kidney, liver, and lung [3,6,7,24,25]. In this context, the protective and preconditioning effects of different VAs regimes, including isoflurane, desflurane, and sevoflurane, have been well studied in both laboratory and experimental animal models as well as in the clinical setting [3,5,6,24]. On the other hand, the effect and administration of VAs in the setting of pancreas transplantation have not yet been well examined.

The aim of this study was to evaluate the effects of different VA regimes on early IRI-associated post-transplant clinical outcomes as well as on short- and long-term allograft function, survival, and (endocrine and metabolic) outcomes in patients who underwent SPKT.

## 2. Methods

### 2.1. Study Design and Study Population

Medical data from all adult patients who underwent SPKT at the University Hospital of Leipzig between 1998 and 2017 were retrospectively analyzed. Our data source compromised a prospectively collected electronic clinical database. In this study, the main focus was placed on the anesthesia and perioperative protocols, as well as the early and long-term allograft function and patient outcomes. The original anesthesia perioperative and operative records were used to determine the usage of the applied anesthetic agents, as well as the perioperative anesthesiological management. Patients younger than 18 years, those receiving kidney transplantation alone (KTA), those receiving pancreatic re-transplantation, and those patients with insufficient/missing data about the perioperative, intraoperative, and postoperative anesthesiological status and outcome were excluded from the study.

### 2.2. Outcome Analysis

Pretransplant standard characteristics of the study population included recipient and donor parameters such as age, sex, body mass index (BMI), donor causes of death, and donor’s comorbidities and clinical course (catecholamine use, creatinine value, arterial hypertension, and intensive care unit lengths of stay (ICU-LOS)). Further, recipient data comprised the duration of diabetes mellitus, time on the waiting list, the duration of pretransplantation dialysis, metabolic endocrine and lipid metabolism, and information on special comorbidities (presence of coronary heart disease, peripheral vascular disease (PVD), blood pressure parameters and arterial hypertension, as well as the number of antihypertensive agents). Peri- and post-transplant data included information on operative and postoperative clinical course, including operation time, blood loss, cold and warm ischemia time of the pancreas and the kidney graft, administered amount and type of intraoperative fluids, amount and type of used catecholamine, and total volumes of blood product transfusions (fresh frozen plasma (FFP), red blood cells).

The occurrence of “clinical IRI” was evaluated, and for the pancreas graft, it was defined as the development of clinically associated outcome parameters and consequences of pancreatic ischemia reperfusion injury, including graft pancreatitis, pancreatic abscess/peritonitis, early delayed graft function, graft thrombosis, rejection, and the consecutive need for re-laparotomy due to graft-related complications within 3 months. In this context, as described previously, the peak of C-reactive protein (CRP, mg/L) and pancreas-specific serum lipase (mmol/L), which were defined as the highest serum levels within the first three days after transplantation, were used as further potential serological pancreatic IRI and graft injury markers [22]. Secondarily, renal graft injury was manifest in the kidney as acute tubular necrosis and DGF/primary nonfunction, with the consequent need for dialysis post-transplant and graft rejection.

Further, immunological and immunosuppressive characteristics (human leukocyte antigen (HLA)- mismatches, cytomegalovirus (CMV)- state, induction therapy), as well as patient outcome and long-term graft function and outcome, were analyzed.

A further focus was placed additionally on the evolution and analysis of cardiovascular events following the SPKT. These included ischemic heart disease documented on a stress test or coronary angiography with or without the need for revascularization. Cerebrovascular accident (CVA) was recorded according to the presence of ischemic or hemorrhagic episodes. Further, peripheral vascular disease (PVD) events were assumed when revascularization or amputation was needed.

Parameters of the endocrine, as well as lipid metabolism low-density lipoprotein (LDL)-cholesterol, high-density lipoprotein (HDL)-cholesterol ratio, HbA1C (%), C-peptide (ng/mL), and renal function variables (Creatinine (mmol/L) and urea (mmol/L)), were analyzed up to five years following transplantation.

Acute rejection episodes were suspected if there were an abrupt increase in serum amylase/lipase or serum glucose levels, together with a significant drop in serum C-peptide level or increased serum creatinine levels and missing diuresis as well as abdominal pain associated with sonographic swelling of the graft. If possible, the diagnosis was confirmed from endoscopic biopsies of the duodenal segment of the graft. Biopsies of the kidney graft were performed to confirm a rejection. Pancreatic biopsies were not performed. Treatment of acute cellular rejection consisted of pulsed steroids or administration of antithymocyte globulin (ATG, 8 mg per kg of body weight) in parallel with increased baseline immunosuppression. DGF of the kidney was defined as the requirement of dialysis in the first week following transplantation [26].

### 2.3. Anesthesia Protocol

All patients were administered general anesthesia via tracheal intubation with invasive mechanical ventilation. General anesthesia was induced with intravenous induction started with an opioid (sufentanyl or fentanyl) after routine preoxygenation, followed by an intravenous anesthetic (propofol or thiopental) as well as the application of a muscle relaxant (rocuronium). General anesthesia was maintained with a halogenated agent (isoflurane, sevoflurane, or desflurane) in an oxygen–air mixture and intermittent application of opioid and muscle relaxants according to the clinical evaluation of the anesthesiologist.

Isoflurane, sevoflurane, and desflurane were used consistently throughout the study period as an individual choice made by the anesthetist.

Additionally, to standard hemodynamic and ventilatory monitoring, direct arterial pressure and central venous pressure were continuously monitored. The arterial catheter was inserted into the radial artery, and the central venous catheter was placed into the right internal jugular vein.

Our center protocol consisted of providing volume repletion and use of vasopressors, as needed, to achieve optimal and appropriate blood pressure at the time of graft reperfusion, ideally a systolic blood pressure level of >140 mmHg/MAP of >70 mmHg. In this context, the volume of fluids administered included crystalloids (mL), fresh-frozen plasma (FFP), human albumin, and transfusion of erythrocyte concentrate. In the case of MAP <70 mmHg, unresponsive to volume repletion, different types of catecholamine were used to maintain the targeted arterial blood pressure levels.

### 2.4. Surgical Techniques and Immunosuppression

As described previously, pancreas and kidney grafts were procured and transplanted following the international standards and guidelines provided by Eurotransplant [27,28,29,30,31,32,33]. In short, the pancreas was explanted in a no-touch technique en-bloc with the spleen and duodenum. After reconstruction of the superior mesenteric and the lineal artery with the donor iliac Y-graft, the pancreas graft was implanted intraperitoneally in the right iliac fossa. The arterial anastomosis was usually performed on the recipient’s common iliac artery, and the venous anastomosis (portal vein) was connected to the inferior vena cava [28,31]. Exocrine drainage was carried out with a hand-sutured side-to-side duodenojejunostomy 40 cm beyond the flexure of Treitz. All kidneys were transplanted into the contralateral iliacal fossa, with vascular anastomoses performed on the external iliac vessels. The ureter was implanted into the bladder according to the Lich–Gregoir technique using a double J catheter as an intraurethral splint [32].

The immunosuppressive protocol consisted of an induction therapy followed by triple maintenance therapy as described previously [32,34]. Shortly, for induction therapy, antithymocyte globulin (Thymoglobulin) or the interleukin-2 receptor antagonist basiliximab (Simulect^®^) was used. Maintenance therapy included calcineurin inhibitors (Cyclosporin (Sandimun Neoral^®^ or Tacrolimus (Prograf^®^), and antimetabolites (Sirolimus (Rapamune^®^), Mycofenolate Mofetil (MMF); (Cell Cept^®^, Myfortic^®^), and tapered steroids (Prednisolone^®^).

### 2.5. Statistical Analysis

With regard to baseline data, continuous variables are illustrated as mean values with standard deviation, and categorical variables are presented as whole numbers and percentages (%). For comparison between the study groups, the appropriate statistical significance test was used, including Student’s *t*-test, χ2, analysis of variance (ANOVA), Kruskal–Wallis and Wilcoxon–Mann–Whitney test.

The primary endpoint of our study was the association and effect of VAs on pancreas allograft failure/survival following SPKT. In this context, pancreas graft failure was defined as resumed insulin therapy, removed pancreas, or re-transplantation. Kidney graft failure was defined as the need for dialysis, removed kidney, or re-transplantation. The secondary endpoint included “IRI-associated post-transplant clinical outcome” as well as long-term graft function and outcome.

A stepwise Cox proportional hazard regression model was used to calculate hazard ratios (HR) with 95% confidence intervals (CI) for assessing pancreas graft failure/survival.

For multivariate analysis, we used a backward regression model including clinically relevant variables and those presenting *p* < 0.05 in univariate analysis.

Survival rates were calculated using Kaplan–Meier method, and a log-rank test was used to test statistical significance between groups. According to previous definitions, graft survival was calculated as the time from initial transplant to graft failure, censoring for death with a functioning graft and grafts still functioning at the time of analysis. Patient survival was defined as the time from transplant to patient death, censoring for patients still alive at the time of analysis. If a recipient was alive or lost to follow-up at the time of the last contact, survival time was censored at the time of the last contact.

All statistical analyses were performed using SPSS software (SPSS Inc., Chicago, IL, USA, version 21.0). A *p*-value <0.05 was considered statistically significant.

“Note: This study has partially analysed data of a prospectively collected database with informations of transplant candidates and transplant recipients after pancreas transplantation. In parts, it has been previously published [11,33,34,35,36,37]. However, these publications considered different inclusion criterias, answered other specific questions (SPKT for type 1 versus type 2 diabetes mellitus, order of graft implantation, type of dialysis modalities etc.) and used further different patient subsets and analyzed different time frames. In this present analysis, we have reported on the effects of different anesthetic agents and perioperative protocols on particular IRI-associated clinical postoperative complications as well as early and long-term graft function and patient outcome. Thus, these aspects and data are not yet published.”

## 3. Results

### 3.1. Baseline Characteristics

During the study period, a total of 105 SPKTs were performed. Of those, 58 patients (55%) received isoflurane, 22 patients (21%) received sevoflurane, and 25 patients (24%) received desflurane as a VA, respectively. The mean follow-up period of the study was 151 ± 34.4 months. Donor and recipients’ demographic and clinicopathologic baseline characteristics according to the three volatile agents used in our study are illustrated in Table 1. Among these groups, significant differences with regard to gender (*p* = 0.03) as well as the number of antihypertensive medications (*p* = 0.03) were recorded. Besides these, no other significant baseline recipient and donor characteristics were observed.

### 3.2. Intraoperative Outcomes and Measurements

Intraoperative outcome parameters of the study group with regard to the VA’s used were described in Table 2. There were no significant differences regarding (intra) operative-related outcome variables nor used fluids/catecholamines between the three different VA groups.

### 3.3. IRI-Associated Clinical Outcome and General Postoperative Outcome

The IRI-associated clinical postoperative outcome, as well as graft function outcome parameters following SPKT stratified by used VA, are shown in Table 3. With regard to pancreas graft injury, rates of graft pancreatitis (isoflurane: 21% versus desflurane: 8% versus sevoflurane: 5%; *p* = 0.04), as well as rates of vascular thrombosis of the pancreas (isoflurane: 14% versus desflurane: 4% versus sevoflurane: 0%; *p* = 0.09), occurred more frequently in the isoflurane group. In addition, there were significant differences in increased early pancreas graft loss within 90 days in the isoflurane group (isoflurane: 24% versus desflurane: 8% versus sevoflurane: 5%; *p* = 0.04). No differences were observed with regard to renal graft injury stated as delayed graft function (*p* = 0.271) and rejection episodes (*p* = 0.228) between the three VA groups.

Further, recipients who received isoflurane had significant higher peak serum CRP and lipase levels (CRP: 143 ± 61 mg/L; lipase: 9.1 ± 6.4 mmol/L) compared with the sevoflurane (CRP: 98 ± 56 mg/L; lipase: 1.95 ± 2.98 mmol/L) and desflurane group (CRP: 112 ± 36 mg/L; lipase: 4.6 ± 7.8 mmol/L; *p* = 0.001 for CRP and *p* = 0.039 for lipase).

General postoperative outcome variables following SPKT were comparable between the three VA groups and showed no significant differences (Supplementary Table S1).

### 3.4. Metabolic Outcome

Concerning metabolic and renal function and outcomes, no significant differences could be found over the follow-up period of 5 years among the three distinct study groups following SPKT (Table 4).

### 3.5. Short- and Long-Term Survival

**Figure 1:**
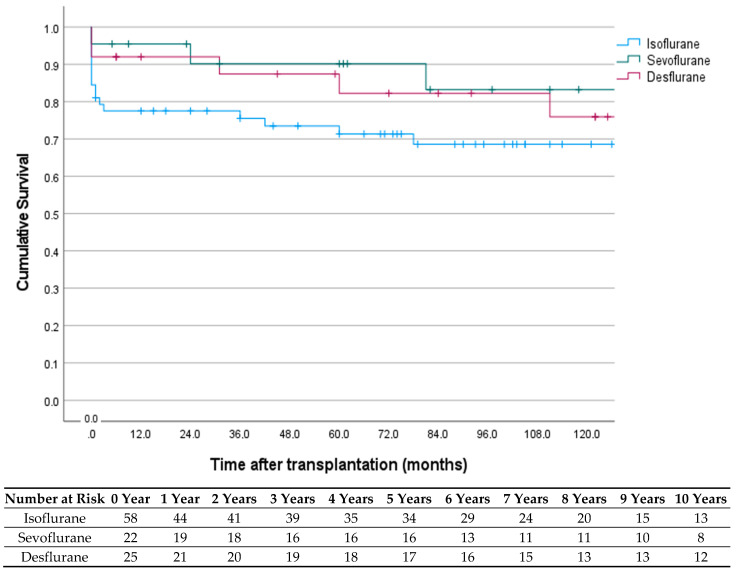
Pancreas graft survival according to the primary inhaled anesthetic agent used at SPKT.

Pancreas graft survival was significantly higher in the sevoflurane and desflurane group compared with the isoflurane group. During the first three months after SPKT, the percentage of pancreas graft loss was 24% in the isoflurane group, 5% in the sevoflurane group, and 8% in the desflurane group (log-rank test: *p* = 0.037). One-, three-, five- and ten-year pancreas graft survival rates in patients after SPKT were 76%, 73.9%, 70%, and 67.1% in the isoflurane group, 90.4%, 85.8%, 78.7, and 78.7% in the sevoflurane group, and 92%, 87.4%, 82.3%, and 75.9% in the desflurane group, respectively (log-rank test: *p* = 0.135 at 10 years) (Figure 1). One-, three-, five-, and ten-year kidney graft survival rates in patients after SPKT were 89.5%, 87.5%, 83.4%, and 76.3% in the isoflurane group, 94.7%, 94.7%, 88.9%, and 88.9% in the sevoflurane group, and 96%, 91%, 81.3%, and 76.7% in the desflurane group, respectively (*p* = 0.746). Overall long-term patient survival at 1, 3, 5, and 10 years was 90%, 87.9%, 83.9%, and 81.5% in the isoflurane group, 95.5%, 95.5%, 89.8%, and 89.8% in the sevoflurane group, and 92%, 92%, 87%, and 76.7% in the desflurane group, respectively (*p* = 0.581).

With regard to cox regression analysis, it could be shown that donor and recipient age, recipient BMI, and the duration of pancreas CIT are independent prognostic predictors of pancreas allograft failure within three months and five years after SPKT. Whereas the choice of VA, recipient gender, donor BMI and gender, transplant era, as well as the order of graft implantation showed a significant impact on pancreas graft survival at three months only, they had no significant effect on the 5-year graft survival (Table 5).

## 4. Discussion

Previous experimental and clinical studies in various organs could demonstrate that anesthetic conditioning, specifically the use of VA, has pleiotropic effects capable of interfering with various pathophysiological pathways that mediate IRI in solid organ transplantation.

Results from our study, which investigated the effects of three different VAs during general anesthesia in patients receiving a simultaneous pancreas and kidney transplantation (SPKT), are in line with previous findings obtained in the kidney [2,7,38], liver [6], and pancreas [9] transplant recipients. Anesthesia with sevoflurane and desflurane demonstrated graft protecting qualities by reducing IRI-associated postoperative complications. Furthermore, serum lipase and CRP levels, both indicators of graft inflammation and injury, were lowest in patients anesthetized with sevoflurane followed by desflurane and isoflurane, resulting in significantly reduced post-transplant pancreatitis and graft vein thrombosis, early after transplantation. These benefits in graft function and survival, however, were short term and could not be determined over the long run, at 10 years after transplantation.

In the course of SPKT, a number of potentially harmful processes inevitably occur, which affect both the viability of the allografts and, subsequently, morbidity and mortality of the graft recipient. Especially in pancreas transplantation, reperfusion injury reflected in elevated post-transplant serum lipase and CRP levels results in severe post-transplant pancreatitis, which consecutively might result in increased rates of graft vein thrombosis, both of which were observed in our isoflurane study group [39,40]. Transplant pancreatitis may not only threaten the graft but can also be associated with major complications such as enteric leak and, worst case, disruption of the vascular anastomosis leading to spontaneous severe hemorrhage with a fatal outcome.

Conditioning is a broad term generally used to describe strategies to attenuate IRI by inducing biochemical changes within the recipient and transplant allograft. Depending on timing and application, it can be referred to as pre-, peri-, and postconditioning. In this context, ischemic preconditioning and remote ischemic preconditioning have shown beneficial effects in solid organ transplantation, including SPKT [37]. This describes the phenomenon whereby brief episodes of ischemia and reperfusion applied in distant tissues, for instance, the lower extremity, render organs subsequently subject to transplantation more resistant to ischemia.

In addition, several pharmacological substances were found to confer tissue tolerance to ischemia by underlying mechanisms similar to those mediating ischemic conditioning. This is also true for several anesthetics, accordingly termed anesthetic conditioning. This is particularly attributed to the volatile anesthetics (VA) used in our study and, to a lesser extent, to intravenous anesthetic agents [41,42]. With regard to the transplant setting, beneficial effects of VAs on IRI-associated consequences and improved graft outcomes could mainly be demonstrated in patients receiving a kidney or liver transplantation, with comparatively small differences between the study groups and also showing relatively short-lived and early improvements [2,6,7,8,38]. More recently, a study by Atoa et al. demonstrated the protective effects of desflurane and sevoflurane compared with isoflurane in the first phase after SPKT, which were in line with our findings [9]. In addition, we were further able to show that there were no abnormalities in endocrine and metabolic function among the groups in the follow-up period. However, the body of evidence concerning protective effects on different organs or the reduction in IRI damage due to anesthetic conditioning using different VAs is conflicting, and not all studies could prove the observed beneficial effects in studies investigating, for instance, tissues of the heart, brain, liver, or kidney [43,44,45,46]. With regard to the transplant setting, the study of Perez-Protto recently failed to show the beneficial effects of VAs application during organ procurement on postoperative graft survival for liver, kidney, lung, or heart transplants [47]. Some other studies also failed to demonstrate the previously observed protective effects of VAs on IRI-associated postoperative complications and graft function and outcome in renal or liver transplant recipients [48,49]. Although the detected beneficial effects of sevoflurane and desflurane compared with isoflurane in IRI were limited to the early phase after transplantation, our findings may be more clinically relevant in the field of pancreas transplantation compared with other forms of solid organ transplantation. It has been shown that ischemia and reperfusion of the pancreas in experimental and clinical pancreas transplantation lead to a profound disturbance of microcirculation and elicits the morphologic and chemical signs of acute pancreatitis within a few hours after reperfusion [10,50]. Serum CRP levels are well evaluated for the assessment of the severity of acute pancreatitis in nontransplant patients [51]. Against this background, peak serum CRP levels during the early period after SPKT might be useful to assess the degree and severity of IRI, specifically postreperfusion pancreatitis in our patients.

However, CRP constitutes a rather unspecific marker of inflammation and infection, which may be elevated due to various infectiological reasons after SPKT and may therefore not be interpreted as a specific marker of IRI or indicator of pancreatic tissue injury. On the other hand, in the early phase, hence in the first 3 days after solid organ transplantation, infections are not very common and therefore do not represent a frequent cause of increased inflammatory parameters. In contrast, post-transplantation pancreatitis is known to cause an increase in CRP during the first few days after SPKT [52]. Therefore, in the absence of other clinical signs and suspicion of early posttransplant infection in the patient, elevated CRP levels within the first 72 hours postoperatively represent a fairly specific marker for pancreatic tissue injury comparable to other forms of acute pancreatitis.

In previous studies, it could be demonstrated that peak CRP levels correlate well with IRI and the consecutive impairment of microcirculation in the early reperfusion period and that an elevation of CRP in the early phase after SPKT is associated with increased pancreas graft-associated complications [22]. Based on these former findings and particularly in combination with the assessment of elevated levels of lipase in our study, we believe that the combination of measurement of peak serum CRP and lipase—besides the clinical evaluation of IRI—may be very useful as further, additional potential biochemical markers of IRI-associated perioperative complications in pancreas transplant recipients.

We would like to highlight the findings in this study of the protective effect of sevoflurane and desflurane compared with isoflurane during general anesthesia in SPKT, which may be of even more direct clinical importance. The use of volatile anesthetics during general anesthesia was associated with reduced IRI damage in various previous studies [3,4,5,6,7,8]. Therefore, VAs seem to be promising agents for the prevention of IRI since they are administered during the whole procedure of transplantation, being applied before, during, and after organ reperfusion, thereby reducing the cellular pathways of IRI induction. However, especially in the light of the rather high accessibility of all three VAs during general anesthesia in transplant centers and relatively few clinically important reasons to choose one VA over the other, any agent which lessens post-transplant pancreas inflammation should therefore be considered for clinical use. In light of higher costs and, for instance, increased ecological and environmental downsides of desflurane, future clinical studies should more precisely evaluate the organ protective effects of sevoflurane according to our promising results in SPKT recipients.

Several limitations of our study are important to discuss. Firstly, the low number of patients in each group and the retrospective nonrandomized design of our current study must be considered. Because of its retrospective design and the rather small number of patients included in the analyses, specifically per analysis group, the results of the study should be interpreted with caution. While direct translation into clinical practice is self-evidently prohibited due to potentially low statistical power and lack of shown causal relation, the purpose and value of retrospective trials are primarily the generation of hypotheses that need to be tested in prospective trials afterward.

Secondly, although this study represents the results of a large German pancreas transplant center with equivalent surgical procedures and compact and robust follow-up data, the long investigation period, as well as different anesthesiologic and operative teams and styles, may have had an impact on the therapeutical decisions, for instance, preferred choice and used VAs in the previous years or different surgical techniques and the order of graft implantation.

Hence, these variables (specifically different analyzed time eras or graft implantation order) could be confounding in a multivariate regression analysis and may also introduce a bias into the observed outcomes in our study.

Therefore, future research should focus on the effects of volatile anesthetics in SPKT and evaluate different dosages of VAs or varying timepoints during the perioperative procedure, as well as undergoing the same surgical graft implantation technique, combined with methods of recipient RIP, over a continuous stable time period.

Alternatively, an interesting strategy for the prevention of IRI may be the evaluation of the effects of the application of different VA dosages and timepoints of exposure of the deceased organ donor to determine the effects of VAs before the onset of ischemia in IRI protection. Unfortunately, in our current study, retrospective data regarding the use of VAs in organ donors could not be obtained.

Moreover, the use of intravenous anesthesia (specifically propofol) may also imply a protective effect against oxidative stress and ischemia–reperfusion injury in major organs. Thus, total intravenous anesthesia is now being popularized in donor and recipient conditioning, with movement away from the VAs at some centers. First insights in the transplant setting with a small patient number and the combination of both agents have already recently shown promising organ protective effects with reduced IRI-associated complications as well as better graft function and outcome in living kidney transplantation [8]. However, the impact of these short-acting intravenous agents on post-transplant IRI has yet to be studied in the clinical setting in future prospective studies with larger patient numbers. This will open a new field of potential combinations of protective intravenous and volatile agents in the prevention of IRI and its complications, as well as the improvement of graft survival and function.

Sevoflurane and desflurane were associated with significantly increased early graft survival as well as decreased IRI-associated post-transplant clinical outcomes when compared with the isoflurane group.

## 5. Conclusions

In our current retrospective study, we found an association between the use of different volatile anesthetics during SPKT and amelioration of IRI with reduced post-transplant morbidity and graft failure. Hereby, we observed a significantly increased early graft survival as well as decreased IRI-associated clinical complications (mainly pancreatitis and graft thrombosis) in the sevoflurane and desflurane groups compared with the isoflurane group. On the other hand, no association between the applied volatile anesthetic and late graft survival and endocrine/metabolic function was observed. Future research in this area with the implementation of prospective, randomized clinical studies should thus focus on the potentially positive effects of different VA agents in patients receiving SPKT and on the investigation of the etiology and underlying cellular mechanisms of VAs and IRI as well as early and long-term graft outcomes. Therefore, administration of VAs at different timepoints and dosages in the deceased organ donor, or administration of the VAs in different dosages and in combinations with intravenous agents as well as in combination with RIP in the recipients during anesthesia for transplantation, might be of great interest.

## Figures and Tables

**Table 1:** Baseline perioperative transplant characteristics of Recipient and Donor according to the primary inhaled anesthetic agent (isoflurane, sevoflurane, desflurane).

Variables	Isoflurane (n = 58)	Sevoflurane (n = 22)	Desflurane (n = 25)	*p*-Value
**Donor**				
Age, years	25.3 ± 11.2	22.0 ± 9.5	22.3 ± 13.2	0.389
Gender, male/ female	30 (47)/24 (53)	5 (26)/14 (74)	6 (29)/15 (71)	0.143
BMI, kg/m^2^	21.5 ± 3.4	22.4 ± 3.5	21.8 ± 4.3	0.597
Catecholamine use	45 (80)	15 (79)	15 (75)	0.880
Stay in the intensive care unit, days	2.7 ± 2.8	3.8 ± 4.1	2.9 ± 4.2	0.398
Creatinine (mmol/L)	81.8 ± 10.9	65.7 ± 8.7	71 ± 6.9	0.500
Hypertension, n (%)	6 (10)	2 (9)	3 (12)	0.947
**Recipient**				
Age, years	43.3 ± 9.2	41.2 ± 10.4	42.1 ± 7.8	0.711
Gender, male/ female	23 (40)/38 (60)	8 (36)/14 (64)	17 (69)/8 (31)	0.030
BMI, kg/m^2^	25.1 ± 4.2	24.8 ± 4.4	24.4 ± 4.2	0.792
HbA1c, (%)	7.9 ± 1.8	7.3 ± 1.7	8.1 ± 1.2	0.386
Duration of Diabetes, years	25.9 ± 7.8	26.2 ± 9.4	28.1 ± 7.9	0.602
Comorbidities				
Cardiovascular disease, n (%)	16 (28)	6 (28)	9 (36)	0.718
Peripheral Vascular Disease, n (%)	10 (18)	3 (14)	4 (16)	0.913
Hypertension, n (%)	46 (79)	19 (86)	19 (76)	0.662
Number of antihypertensive medications	2.2 ± 1.9	2.9 ±1.5	2.0 ± 1.6	0.030
Previous Dialysis, n (%)	46 (79)	17 (77)	20 (80)	0.971
Duration of dialysis, months	31.1 ± 3.7	36.2 ± 8.3	23.5 ± 5.2	0.421
Waiting time, months	11.7 ± 12.4	8.2 ± 13.8	6.2 ± 1.5	0.204
**Transplant characteristics**				
CMV D+/R−	12 (21)	4 (18)	4 (16)	0.876
HLA Mismatches > 2/6	46 (79)	13 (60)	16 (64)	
Immunosuppression				
Induction therapy (ATG/IL-2 RA/None)	36/15/7 (62/26/12)	14/5/3 (64/23/13)	19/5/1 (76/204)	0.708
CNI, tacrolimus/ CsA	53/5 (91/9)	19/3 (86/14)	24/1 (96/4)	0.500
AP drug, MMF/ SRL /none	51/4/3 (88/7/5)	17/5/0 (78/23/0)	19/6/0 (76/24/0)	0.099

Table legends: BMI—body mass index; Hb1Ac—glycosylated hemoglobin; CMV—cytomegalovirus; HLA—human leukocyte antigen; ATG—antithymocyte globulin; IL-2 RA—interleukin-2 receptor antagonist; CNI—calcineurin inhibitor; CsA—cyclosporin A; AP drug—antiproliferative drug; MMF—mycophenolate mofetil; SRL—sirolimus.

**Table 2:** Intraoperative outcome and measurements according to the primary inhaled anesthetic agent (isoflurane, sevoflurane, desflurane).

Variables	Isoflurane (n = 58)	Sevoflurane (n = 22)	Desflurane (n = 25)	*p*-Value
Cold ischemia time, hours				
Pancreas	11.1 ± 2.9	11.8 ± 2.6	10.5 ± 2.1	0.253
Kidney	11.7 ± 2.4	12.4 ± 3.9	11.4 ± 2.8	0.545
Warm ischemia time, minutes				
Pancreas	39.1 ± 2.9	37.7 ± 2.7	36.1 ± 1.8	0.440
Kidney	36.3 ± 1.7	38.9 ± 2.6	36.8 ± 2.1	0.724
Operating time, minutes	381 ± 11	367 ± 20	399 ± 28	0.380
Intravenous infusions, mL				
Total amount	4100 ± 1859	3789 ± 1179	4320 ± 1520	0.189
Type of fluids				
Crystalloids	19 (33)	9 (41)	8 (32)	0.761
Combination Crystalloids/Colloids	39 (67)	13 (59)	17 (68)	
Type of catecholamine				
Norepinephrine	13 (22)	5 (23)	3 (12)	0.521
Dopamin	8 (14)	4 (18)	6 (24)	
Akrinor	9 (16)	2 (9)	3 (12)	
Dobutamine	3 (5)	1 (7)	1 (4)	
Combination of norepinephrine/dobutamine	15 (26)	3 (14)	2 (8)	
Combination norepinephrine/dobutamine/epinephrine	4 (7)	2 (9)	2 (8)	
Epinephrine	3 (5)	2 (9)	2 (8)	
Combination norepinephrine/akrinor	3 (5)	3 (14)	6 (24)	
Total Transfusion, mL				
Red blood cell	314 ± 56	200 ± 105	436 ± 137	0.325
Fresh-frozen plasma (FFP)	183 ± 45	95 ± 32	204 ± 93	0.359
Blood loss (mL)	1050 ± 156	950 ± 240	1100 ± 160	0.567

Table legends: FFP, fresh frozen plasma.

**Table 3:** Ischemia–reperfusion injury-associated post-transplant clinical outcome parameters following simultaneous pancreas–kidney transplantation stratified by the primary inhaled anesthetic agent (isoflurane, sevoflurane, and desflurane).

Variables	Isoflurane (n = 58)	Sevoflurane (n = 22)	Desflurane (n = 25)	*p*-Value
Vascular thrombosis pancreas (%)	8 (14)	0	1 (4)	0.090
Combined acute rejection episodes (%)	10 (17)	1 (5)	2 (8)	0.228
Pancreatitis (%)	12 (21)	1 (5)	2 (8)	0.040
Peak lipase, (mmol/L)	9.1 ± 6.4	1.95 ± 2.98	4.6 ± 7.8	0.039
Peak CRP, (mg/L)	143 ± 61	98 ± 56	112 ± 36	0.001
Delayed renal graft function (%)	13 (22)	2 (9)	3 (12)	0.271

**Table 4:** Metabolic outcome after simultaneous pancreas–kidney transplantation stratified by the primary inhaled anesthetic agent (isoflurane, sevoflurane, desflurane).

Variables	Time after SPKT															
	6 Months				1 Year				3 Year				5 Years			
	Iso-Flurane	Sevo-Flurane	Des-Flurane	*p*-Value	Iso-Flurane	Sevo-Flurane	Des-Flurane	*p*-Value	Iso-Flurane	Sevo-Flurane	Des-Flurane	*p*-Value	Iso-Flurane	Sevo-Flurane	Des-Flurane	*p*-Value
C-peptide, ng/mL	2.3 ± 1.9	2.9 ± 1.7	2.6 ± 1.5	0.456	2.5 ± 1.3	1.5 ± 0.8	1.2 ± 0.6	0.801	1.1 ± 0.6	1.0 ± 0.2	0.9 ± 0.8	0.804	0.9 ± 0.6	0.8 ± 0.6	1.1 ± 0.1	0.789
HbA1c, %	5.7 ± 0.8	5.6 ± 0.8	5.9 ± 1.3	0.753	5.7 ± 1.1	5.4 ± 0.5	5.6 ± 0.6	0.678	5.5 ± 0.8	5.2 ± 0.4	5.7 ± 1.3	0.456	5.9 ± 1.2	5.3 ± 0.6	6.1 ± 2.4	0.109
Creatine, mmol/L	125 ± 54	126 ± 37	131 ± 54	0.987	115 ± 29	110 ± 45	145 ± 28	0.277	135 ± 12	104 ± 23	125 ± 34	0.145	122 ± 53	110 ± 25	145 ± 32	0.267
Urea, mmol/L	8.8 ± 4.5	7.9 ± 3.1	8.1 ± 3.3	0.706	10.2 ±6.5	8.2 ± 3.9	11.5 ± 29	0.876	11.1 ± 8.4	8.3 ± 0.6	9.2 ± 5.1	0.245	8.9 ± 3.2	6.3 ± 4.9	11.5 ± 6.2	0.152
LDL/HDL- cholesterol ratio	2.1 ± 0.9	1.9 ± 0.7	1.9 ± 0.7	0.465	1.85 ± 0.9	1.8 ± 1.0	2.1 ± 1.1	0.779	1.9 ± 1.1	1.6± 0.5	1.9 ± 0.7	0.601	2.0 ± 0.8	1.9 ± 0.9	2.1 ± 0.9	0.233

Table legends: HbA1c—glycosylated hemoglobin; LDL/HDL—low-density lipoprotein/high-density lipoprotein.

**Table 5:** Logistic regression analysis of predictors for pancreas allograft failure following simultaneous pancreas-kidney transplantation.

Variables				Time after SPKT											
				3 Months						5 Years					
				Univariate Analysis			Multivariate Analysis			Univariate Analysis			Multivariate Analysis		
				HR	95% CI	*p*-Value	HR	95 CI	*p*-Value	HR	95 CI	*p*-Value	HR	95 CI	*p*-Value
**Donor**															
	Age *			1.09	1.02–1.13	0.002	1.05	1.01–1.98	0.012	1.06	1.02–1.09	0.003	1.061	1.03–1.11	0.001
	Gender (male vs. female)			1.37	0.58–3.25	0.251				3.7	1.02–8.45	0.145			
	BMI *			1.16	1.02–1.35	0.032	1.24	1.07–1.42	0.003	1.16	1.02–1.35	0.026	1.11	0.92–1.31	0.174
**Recipient**															
	Age *			1.06	1.01–1.13	0.013	1.10	1.03–1.18	0.004	1.08	1.02–1.14	0.008	1.06	1.011–1.12	0.018
	Gender (male vs. female) *			0.33	0.15–0.97	0.036	0.24	0.08–0.70	0.008	0.58	0.25–1.31	0.07			
	BMI *			1.17	1.06–1.31	0.001	1.23	1.09–1.39	0.008	1.20	1.01–1.35	<0.001	1.26	1.06–1.41	0.005
**Transplant**															
	Era (1998–2006 vs. 2007–2017) *			4.8	1.1–21.14	0.035	7.1	1.5–33.5	0.013	2.11	0.86–625	0.089			
	Implantation order graft			3.15	1.05–9.50	0.040	4.17	1.35–12.85	0.013	2.09	0.82–5.29	0.110			
	(pancreas first vs. kidney first) *														
	Warm ischemia time														
		Pancreas		0.996	0.94–1.07	0.821				0.88	0.25–1.97	0.453			
		Kidney		1.03	0.98–1.09	0.231				0.99	0.95–1.08	0.856			
	CIT, hours														
		Pancreas *													
			0–8	Ref		0.002	Ref		0.004	Ref		0.02	Ref		0.06
			8–12	0.61	0.1–12.4	0.129	0.58	0.05–0.86	0.01	5.18	0.61–43.4	0.131	2.98	0.6–14.9	0.183
			>12	3.7	1.1–13.1	0.04	8.5	1.3–114.9	0.02	11.3	1.5–86.3	0.019	5.38	1.21–23.7	0.027
		Kidney *													
			0–8	Ref		0.07				Ref		0.012	Ref		0.008
			8–12	0.46	0.2–8.9	0.58				0.13	0.02–0.99	0.048	0.38	0.1–1.6	0.07
			>12	3.89	0.21–34.8	0.18				1.82	0.33–8.02	0.164	1.1	0.82–8.8	0.451
	Volatile Anesthetics *														
		Isoflurane		Ref		0.037	Ref		0.020	Ref		0.07			
		Sevoflurane		0.13	0.02–0.98	0.048	0.12	0.02–0.93	0.033	0.13	0.2–0.99	0.051			
		Desflurane		0.23	0.05–1.01	0.051	0.28	0.06–1.07	0.060	0.46	0.15–1.37	0.167			
Immunosuppression															
	Induction therapy														
		None		Ref.		0.791				Ref.		0.342			
		ATG		0.63	0.16–2.58	0.527				0.78	0.21–2.96	0.722			
		IL-2 RA		0.72	0.19–2.91	0.791				1.03	0.27–4.12	0.961			

Table legends: BMI—body mass index; CIT—cold ischemia time; ATG—antithymoctye globulin; IL-2 RA—interleukin-2 receptor antagonist. * included in multivariate analysis.

## Data Availability

Our database contains highly sensitive data that may reveal clinical and personnel information about our patients and lead to their identification. Therefore, according to organizational restrictions and regulations, these data cannot be made publicly available. However, the datasets used and analyzed in the current study are available from the corresponding author upon reasonable request.

## Supplementary Materials

The following supporting information can be downloaded at: https://www.mdpi.com/article/10.3390/jcm11123385/s1, Table S1: General postoperative outcome following simultaneous pancreas-kidney transplantation stratified by the primary inhaled anesthetic agent (isoflurane, sevoflurane, desflurane).

## Abbreviations

ANOVA	Analysis of variance
ARE	Acute rejection episode
ATG	Antithymoctye globulin
BMI	Body mass index
CI	Confidence interval
CMV	Cytomegalovirus
CNI	Calcineurin inhibitor
CRP	C-reactive protein
CVA	Cerebrovascular accident
DGF	Delayed graft function
DSO	Deutsche Stiftung Organtransplantation
FFP	Fresh frozen plasma
HDL	High-density lipoprotein
HLA	Human leukocyte antigen
HR	Hazard ratio
IL2-RA	Interleukin-receptor-antagonist
IRI	Ischemia reperfusion injury
KTA	Kidney transplant alone
LDL	Low-density lipoprotein
MMF	Mycophenolate mofetil
PVD	Peripheral vascular disease
SPKT	Simultaneous pancreas–kidney transplantation
SRL	Sirolimus
VA	Volatile Anesthetics
