# Supplementary material for: Analysis of Volatile Anesthetic-Induced Organ Protection in Simultaneous Pancreas–Kidney Transplantation
Source: J Clin Med. 2022 Jun 13;11(12):3385. doi: 10.3390/jcm11123385 (PMC9225086; doi:10.3390/jcm11123385)
Supplement: Supplementary file 1 [file jcm-11-03385-s001.zip › jcm-1634832-supplementary.pdf]

Supplementary Table S1: General postoperative outcome following simultaneous pancreas-kidney transplantation stratified by the primary inhaled anesthetic agent (isoflurane, sevoflurane, desflurane)

Variables	Isoflurane (n = 58)	Sevoflurane (n = 22)	Desflurane (n = 25)	P-value
Cardiovascular events	15 (14.2)	2 (9.1)	8 (32)	0.151
Re-operation rates (%)	25 (43)	5 (22)	7 (28)	0.167
Bleeding (%)	6 (10)	1 (4)	2 (8)	0.705
Hospital stay, days	47.9 +/- 29.3	38.2 +/- 19.5	41.3 +/- 13.2	0.219
CMV Infection (%)	14 (24)	3 (13)	4 (16)	0.489
